# Pharmacokinetic and pharmacodynamic studies of CD19 CAR T cell in human leukaemic xenograft models with dual‐modality imaging

**DOI:** 10.1111/jcmm.16776

**Published:** 2021-07-09

**Authors:** Qiong Wu, Yan Wang, Xinyu Wang, Ningxia Liang, Jingjing Liu, Donghui Pan, Yuping Xu, Lizhen Wang, Junjie Yan, Guangji Wang, Liyan Miao, Min Yang

**Affiliations:** ^1^ First School of Clinical Medicine Nanjing Medical University Nanjing China; ^2^ NHC Key Laboratory of Nuclear Medicine Jiangsu Key Laboratory of Molecular Nuclear Medicine Jiangsu Institute of Nuclear Medicine Wuxi China; ^3^ Department of Clinical Pharmacology The First Affiliated Hospital of Soochow University Suzhou China; ^4^ Institute for Interdisciplinary Drug Research and Translational Sciences College of Pharmaceutical Sciences Soochow University Suzhou China; ^5^ Key Laboratory of Drug Metabolism and Pharmacokinetics State Key Laboratory of Natural Medicines China Pharmaceutical University Nanjing China

**Keywords:** CD19 CAR T cell, pharmacodynamics, pharmacokinetics, positron emission tomography, solid tumour

## Abstract

In recent years, chimeric antigen receptor T (CAR T)‐cell therapy has shown great potential in treating haematologic disease, but no breakthrough has been achieved in solid tumours. In order to clarify the antitumour mechanism of CAR T cell in solid tumours, the pharmacokinetic (PK) and pharmacodynamic (PD) investigations of CD19 CAR T cell were performed in human leukaemic xenograft mouse models. For PK investigation, we radiolabelled CD19 CAR T cell with ^89^Zr and used PET imaging in the CD19‐positive and the CD19‐negative K562‐luc animal models. For PD evaluation, optical imaging, tumour volume measurement and DNA copy‐number detection were performed. Unfortunately, the qPCR results of the DNA copy number in the blood were below the detection limit. The tumour‐specific uptake was higher in the CD19‐positive model than in the CD19‐negative model, and this was consistent with the PD results. The preliminary PK and PD studies of CD19 CAR T cell in solid tumours are instructive. Considering the less efficiency of CAR T‐cell therapy of solid tumours with the limited number of CAR T cells entering the interior of solid tumours, this study is suggestive for the subsequent CAR T‐cell design and evaluation of solid tumour therapy.

## 1 INTRODUCTION

Currently, there are 468 targets involved in the development of 4,000 immunotherapy drugs worldwide.^1^ Among them, CD19 targets are always at the top of the research hot spots (192), and drugs targeting CD19 are already on the market.^2^ CD19 is a type of leucocyte differentiation antigen, an important membrane antigen related to B‐cell proliferation, differentiation, activation and antibody production, and is the best marker for diagnosing B‐cell lineage tumours (leukaemia and lymphoma) and identifying B cells.^3^ Chimeric antigen receptor (CAR) T therapy is a new type of cellular therapy technology, with special clinical results against haematoma.^4^ At present, about half of the CAR T‐cell targets are CD19, and the two CD19 CAR T products, Kymriah^5^ and Yescarta,^6^ have been approved for marketing by FDA (Food and Drug Administration) and EMA (European Medicines Agency). CD19 is the most frequent antigen target in the CAR T clinical projects accepted by the National Medical Products Administration (NMPA) in China.^7^ Currently, CAR T cells have made some breakthroughs in the treatment of haematological tumours, but reports of solid tumours targeting CD19 are still rare.

CAR T cells are effective in treating haematologic malignancy directly in contact with blood tumour cells.^8^ The efficacy of CAR T therapy is closely related to the number and activity of CAR T cells for the haematoma patients. While for the solid tumours, CAR T cells need to be transferred from peripheral blood to interior solid tumour and traverse the solid masses formed in the early stage to exert antitumour effects, the number and activity of CAR T cells migrating to solid tumour tissues are greatly reduced.^9^ In addition, another major challenge is the selection of target because tumour tissue is heterogeneous and multiple target antigens may be expressed in the same tumour.^10^ Moreover, there are also some adverse effects during the CAR T‐cell therapy.^11^ To meet these challenges, there is an urgent need to develop a sensitive, real‐time, continuous, comprehensive and accurate visualization technique to reveal the distribution, migration and targeting of CD19 CAR T cells in vivo.^12^, ^13^ Current clinical monitoring of the efficacy of CAR T therapy includes tumour biopsy and DNA copy number in blood.^14^, ^15^ Tissue biopsies are difficult for some patients, and the sample may not represent accurate result due to the heterogeneity within the solid tumour.^16^ DNA copy number in blood is currently not very sensitive, and the indicators in blood and bone marrow do not correlate with the infiltration of tumour masses.^17^, ^18^ The lack of monitoring methods of CAR T cells’ biological behaviour in vivo has greatly limited its development and application.

Molecular imaging techniques, such as magnetic resonance imaging (MRI), single‐photon emission computerized tomography (SPECT), positron emission tomography (PET) and fluorescent imaging, which can provide non‐invasive, reproducible and quantitative tracking of implanted cells, might elucidate the above problems.^19^ After labelled with superparamagnetic iron oxide nanoparticle (SPIO) agents, MRI can be used to monitor the SPIO‐labelled stem cells from the injection site to the infarct area.^20^ However, it is difficult to achieve whole‐body imaging of the distribution of SPIO‐labelled cells by MRI, as the dark signal induced by SPIOs may also be derived from other sources.^21^ ^111^In‐oxine labelling is always a gold standard approach for cell tracking in vivo by scintigraphy or SPECT. However, this method requires relatively high levels of radioactivity, which might induce cellular damage.^22^ In comparison with SPECT, PET has higher resolution and higher sensitivity,^23^ making positron nuclide labelling an attractive choice for PET imaging cells. PET has been reported to track infused cells^24^ with radionuclides ^89^Zr, ^68^Ga and ^52^Mn^23^, ^25^, ^26^, ^27^ Previously, we have successfully used ^89^Zr‐ and ^68^Ga‐labelled CAR T cells to obtain early in vivo distribution and migration behaviour, but has not yet demonstrated its targeting and efficacy in solid tumours.^27^

Here, in order to verify the targeting and effectiveness of CD19 CAR T cells in CD19 high expression leukaemic solid tumours, we used ^89^Zr as the radionuclide to label CAR T cells, and then, PET and optical imaging were used to investigate the in vivo PK and PD of CAR T cells.

## 2 MATERIALS AND METHODS

### 2.1 Materials

8‐Hydroxyquinoline (oxine), Na_2_CO_3_ and 2‐[4‐(2‐hydroxyethyl) piperazin‐1‐yl] ethane sulphonic acid (HEPES) solution were purchased from Sigma‐Aldrich (St Louis, MO). Dimethyl sulphoxide (DMSO) was purchased from Acros Organics (Belgium, USA). D‐Luciferin was purchased from PerkinElmer (Massachusetts, USA). ^89^Zr‐oxalate was obtained from Dongcheng AMS Pharmaceutical Co., Ltd (Nanjing China).

The human chronic myelogenous leukaemic cell line K562 was originally purchased from ATCC (Manassas, VA). A lentiviral vector that encoded the human CD19 gene and a firefly luciferase reporter gene was transduced into the K562 cell line to generate the CD19‐K562‐luc cell line as a CD19‐positive cell. Another lentiviral vector that encoded a firefly luciferase reporter gene was transduced into the K562 cell line to generate the K562‐luc cell line as a CD19‐negative cell. The CD19‐positive cell and CD19‐negative cell were cultured at 37℃ with 5% CO_2_ in ATCC modified RPMI 1640 medium (Gibco) containing 10% foetal bovine serum (Gibco) and 1% penicillin‐streptomycin. CD19 CAR T cells were gifted from Shanghai Unicar Biomed‐Pharmaceutical Technology Co., Ltd.

### 2.2 Targeting of CD19 CAR T cells

CD19 CAR T cells were generated as described by LiQing Kang et al.^18^ To verify in vitro targeting validation of CD19 CAR T cells, we conducted cytotoxicity assays using the Cytotoxicity Detection Kit (Promega, Madison, WI, USA) following the manufacturer's protocol. All the transduced CAR T cells (effector, E) were co‐cultured with cells expressing CD19 (target, T) at E:T ratios of 10:1, 5:1 and 2.5:1, respectively. Target and effector cells were seeded into 96‐well plates in a total volume of 100 μL of serum‐free RPMI 1640 media (Gibco) and incubated at 37℃ for 16 hours. After co‐culture, 50 μL of cell‐free supernatant from each well was transferred to the new 96‐well plates and mixed with an equal volume of lactic acid dehydrogenase substrate mixture for 20 minutes at room temperature in the dark. The absorbance was recorded at 492 nm using a full wavelength reader Multiskan GO (Thermo Scientific). Tumour (target cell) lysis was calculated with the following formula: % lysis = (experimental LDH release − spontaneous LDH release)/(maximum LDH release − spontaneous LDH release) × 100%.

In addition, we performed antigen stimulation of CAR T cells and T cells with CD19‐positive and CD19‐negative cells, respectively, and examined cytokine release and cell proliferation after antigen stimulation. Cytokine release was evaluated using a Th1/Th2 Cytometric Bead Array (CBA) Kit II (BD Bioscience) according to the manufacturer's instructions. Briefly, CAR‐transduced T cells or non‐transduced T cells were co‐cultured with the CD19‐positive/negative cell at an E:T ratio of 5:1 in a 96‐well plate in a total volume of 200 μL of RPMI 1640 medium (Gibco). After 24‐h co‐culture, cell‐free supernatants were harvested and the levels of various cytokines were evaluated. The capture microspheres for seven specific cytokines (IL‐2, IL‐4, IL‐6, IL‐10, IFN‐γ, TNF‐a and IL‐17A) were first mixed and then incubated with the sample and fluorescent antibody for 3 hours. The mixture was washed, and cytokine concentrations were determined by flow cytometry (Thermo Fisher). The concentration of each cytokine was calculated from standard curves.

Cell proliferation assays were performed using a carboxyfluorescein diacetate succinimidyl ester (CFSE) assay kit (Abcam, Cambridge, UK) following the manufacturer's instructions. In brief, the CAR T cells were labelled with 2.5 μM CFSE and then co‐cultured with CD19‐positive/negative cells, which treated with mitomycin before to stop the division, at a stimulator‐to‐responder ratio of 5:1 (10^6^ CAR T cells/mL) for 5 days in 24‐well plates in 500 μL serum‐free AIMV (Gibco) medium per well. Flow cytometry was performed using an Attune NxT flow cytometer (Thermo Fisher) to detect changes in CFSE intensity. FlowJo v10 software (TreeStar, San Carlos, CA, USA) was used for data analysis.

### 2.3 Human leukaemic xenograft models

Thirty‐one female NOD/ShiLtJGpt‐Prkdcem26Cd52Il2rgem26Cd22/Gpt (NCG, NBRI) mice aged 6 weeks were purchased from GemPharmatech Co., Ltd. For the localized disease model, mice were inoculated subcutaneously (s.c.) on the right leg with 5 × 10^6^ CD19‐K562‐luc cells. All animal experiments were approved by the Institutional Animal Care and Ethics Committee of Jiangsu Institute of Nuclear Medicine (Wuxi, China).

### 2.4 Radiolabelling of CAR T cells

CD19 CAR T cells were radiolabelled with ^89^Zr using methods reported before with some modifications.^27^ Briefly, we adjusted the pH of the ^89^Zr^4+^ solution to 7 with 1 M Na_2_CO_3_ and 0.1 M HEPES buffer. 20 μg oxine dissolved in 20 μL 10% acetic acid was then added to the ^89^Zr^4+^ solution and reacted for 15 minutes at room temperature. For radiolabelled CAR T cells, 2*10^6^ CAR T cells were incubated with 3 MBq ^89^Zr‐oxine solution for 10 min at room temperature and then centrifuged at 2000 rpm for 5 min. Finally, the ^89^Zr‐CAR T cells were used for injection after washing three times with saline.

### 2.5 Group and treatment

The mice were then randomly divided into four groups as shown in Table 1. Group 1 (n = 12) and group 3 (n = 4) were the CD19‐positive xenograft model. Group 2 (n = 12) and group 4 (n = 3) were the CD19‐negative xenograft model. Group 1 and group 2 were treated with 2*10^6 89^Zr‐labelled CAR T via tail vein. Group 3 and group 4 were treated with saline.

**TABLE 1:** Experimental groups and treatment protocols

Group	Model	Animal Numbers	Administration
1 (treated)	CD19 positive	12	2*10^6^ CAR T/approx. 3 MBq
2 (treated)	CD19 negative	12	2*10^6^ CAR T/approx. 3 MBq
3 (control)	CD19 positive	4	Null
4 (control)	CD19 negative	3	Null

### 2.6 Pharmacodynamic study

To evaluate the therapeutic efficacy of CD19 CAR T cells in human leukaemic xenograft models, CD19‐positive model and CD19‐negative model mice were employed and received CD19 CAR T therapy. The tumour volume and bodyweight were monitored every two days for 25 days, as well as the state and survival of the animal models. Furthermore, the cell activity of xenograft tumour was evaluated using in vivo fluorescent imaging with digital callipers and IVIS spectrum imaging system (PerkinElmer, Inc). Mice were received 15 mg/mL D‐luciferin working solution through intraperitoneal injection and were anaesthetized after 5 minutes of injection, and image analysis was performed after 10 minutes of injection. The mice were scheduled for optical imaging on days 2, 4, 6, 8, 10 and 13. Based on our previous exploration, we set the exposure time of fluorescent image as 30 s and the fluorescent wavelength as 560 nm. The images were analysed by Living Image® software (Xenogen, CA).

Six animals randomly selected from the G1 and G2 groups were killed at 168 hours for blood sample collection, and the rest of each group were continuously monitored with PET and optical imaging. Each blood sample was approximately 200 μL. DNA was extracted from the blood sample using the TIANamp Blood DNA Kit (Tiangen, China) according to the kit instructions, and then amplified with primers and probes complementary to specific sequences within the lentiviral vector. A gradient dilution of the plasmid encoding the CAR gene was used, and a standard curve was established using the gradient dilution plasmid solution. Amplification was performed using an ABI 7500 Real‐Time PCR System (Applied Biosystems, Thermo Fisher Scientific, USA), and data were analysed using software that comes with the instrument.

### 2.7 micro‐PET Image

In order to monitor the in vivo distribution of CD19 CAR T cells, PET scans were performed on an Inveon scanner (Siemens Medical Solutions, Germany). The mice were anaesthetized with 1.5%‐2.5% isoflurane/oxygen mixture before and during the scanning. CD19‐positive model and CD19‐negative model mice were intravenously injected with 2*10^6 89^Zr‐CAR T cells via the tail vein. The radioactivity of each mouse was 2.96 MBq. For ^89^Zr‐CAR T‐cell imaging, the 10‐min‐ or 20‐min‐long static PET scans were performed at 2, 4, 6, 24, 48, 72, 90, 112, 140, 168 and 260 h p.i.

PET images were reconstructed using a 3‐dimensional ordered subset expectation maximum algorithm (OSEM‐3D), and the image data were analysed by ASIPro software. The major organs and tissues of each mouse, such as the lung, liver, tumour and spleen, were delineated manually on the images as the regions of interest (ROIs). The image‐derived percentage of injected dose per gram (%ID/g) was calculated for each ROI and used as the indicator for the quantification of radioactivity uptake. The %ID/g can be obtained using the following equations:

$$\%\text{ID}/g=\frac{\text{ROI activity concentration}(\text{KBq}/g)}{\text{Total injected activity}(\text{KBq})}*100$$

In order to obtain the kinetics of ^89^Zr‐CAR T‐cell distribution in vivo, we mapped the uptake curve of four main organs (lung, liver, spleen and tumour) with representative mean %ID/g.

### 2.8 Ex vivo biodistribution and blood pharmacokinetics

Six CD19‐positive model and six CD19‐negative model mice were randomly selected from treated groups for biodistribution and pharmacokinetic studies. Each model mouse was intravenously administrated with 2*10^6 89^Zr‐CAR T cells. Blood samples were collected from the tail (10‐20 µL per animal) at 5 min, 10 min, 15 min, 30 min, 1 h, 2 h, 4 h, 6 h, 24 h, 48 h, 72 h and 96 h after injection, weighed immediately and detected with a gamma counter (2480 WIZARD2; PerkinElmer). The uptake (%ID/g) of blood was then calculated by the following equation:

$$\text{blood uptake}\left(\%\text{ID}/g\right)=\text{bloodcounter}\left(\text{CPM}\right)/\text{whole ‐ body counter}\left(\text{CPM}\right)/\text{blood sample weight}*100$$

Pharmacokinetic parameters, such as AUC and half‐time (*t*_1/2_), were then fitted with DAS (version 1.1) software.^28^

After killing the mice on day 7, blood and organs (brain, heart, liver, spleen, lung, kidney, stomach, duodenum, colon, muscle, sexual gland, fat, tibia, joint, marrow, adrenal gland, gladder and tumour) were collected, weighed immediately and γ‐counted together with standards prepared from a sample of injected material. The percentage of injected dose per gram (% ID/g) of tissue was calculated by the following equation:

$$\text{Tissue uptake}\left(\%\text{ID}/g\right)=\text{tissue counter}\left(\text{CPM}\right)/\text{whole ‐ body counter}\left(\text{CPM}\right)/\text{tissue sample weight}*100$$

### 2.9 Specific tumour uptake

Referring to the previous study,^29^ the tumour uptake of ^89^Zr PET imaging consists of two parts: one was the specific part, which related to the cumulative exposure of ^89^Zr‐CAR T cells over time; and the other was the non‐specific part, which associated with the plasma concentration (Cp). The Patlak model: $\mathrm{Tumour}\;\mathrm{uptake}\;\left(\%\mathrm{ID}/g\right)=A*\mathrm{Cp}\left(t\right)+B*\int_{0}^{t}\mathrm{Cp}\left(\tau \right)d\tau$ can be used to quantify the specific and non‐specific parts. Here, the A value is associated with non‐target binding components, while the B‐value can reflect the degree of specific binding.

In view of the strong correlation between blood samples taken from tail and heart through PET imaging,^29^ and moreover, the data from tail blood are more accurate and reliable than heart blood in this study, the blood activities from tail were used as the input functions to establish the Patlak model. In addition, the B‐value was used as a key parameter for the tumour‐specific uptake of CD19 CAR T cells in CD19‐positive tumours.

### 2.10 Statistical analysis

Results are expressed as the mean ± SD unless stated otherwise. Statistical comparisons between two groups were evaluated with Student's *t* test (unpaired *t* test, two‐tailed). A probability (*P*) value of .05 was considered to indicate statistical significance. Statistical analysis was performed with GraphPad Prism 7, and figures were plotted with GraphPad Prism 7.

## 3 RESULTS

### 3.1 In vitro targeting validation of CD19 CAR T cells

**FIGURE 1:**
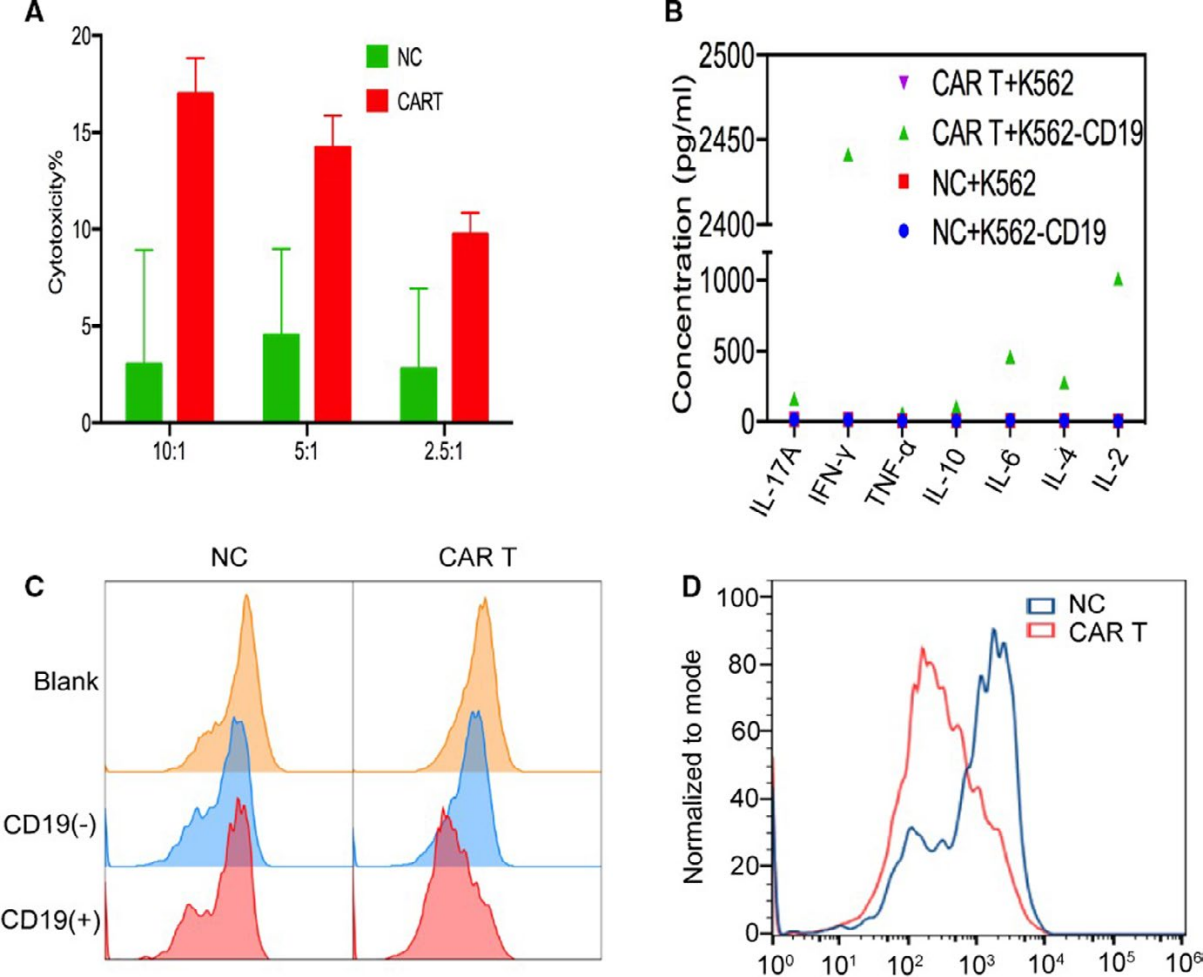
In vitro targeting validation of CD19 CAR T cells. A, T cells (NC group) and CD19 CAR T cells were examined using the LDH method to verify the killing function. B, The cytokine secretion function was assessed by flow cytometry to detect the concentrations of IL‐17A, IFN‐γ, TNF‐α, IL‐10, IL‐6, IL‐4 and IL‐2. C, Cell proliferation of NC and CAR T cells was detected by CFSE staining. D, The CD19(+) peak plots of NC and CAR T cells were superimposed

To verify the killing function of CD19 CAR T, T cells (normal control group, abbreviated as NC group) and CD19 CAR T cells were examined using the LDH method. The results showed that the killing efficiency of CAR T on tumour cells was significantly higher than that of the T cells after co‐incubation at potent target ratios of 10:1, 5:1 and 2.5:1 (Figure 1A), indicating that CD19 CAR T cells killed CD19 antigen‐positive tumour cells in a dose‐dependent manner. The cytokine secretion function was assessed by E:T = 5:1 co‐incubation (Figure 1B), and the incubation supernatants were collected to detect the concentrations of IL‐17A, IFN‐γ, TNF‐α, IL‐10, IL‐6, IL‐4 and IL‐2 by flow cytometry. The graph of CAR T and K562‐CD19 co‐incubation group showed a significant increase in factor concentration, which indicated that CAR T cells can be stimulated by target cells to produce cytokines. The results of cell proliferation detection by CFSE staining are shown in Figure 1C,D. Among the fluorescent intensity peak plots of Blank, K562 and K562‐CD19 subgroups of CAR T cells, the peak plot of the K562‐CD19 group showed a significant left shift, suggesting that CAR T cells proliferated, while the T cells did not. Furthermore, fluorescent intensity peak plots of its three subgroups were similar, reflecting the cells did not proliferate significantly. In addition, the K562‐CD19 peak plots of NC and CAR T cells were superimposed in Figure 1D. The fluorescence of CFSE was highly attenuated, which indicates that CD19‐K562‐luc cells can specifically stimulate CD19 CAR T cells to induce proliferation.

### 3.2 Radiolabelling of CAR T cells

Consistent with the results of our previous study,^27^ the cell viability (>90%) and ^89^Zr retention (>80%) of the CAR T cells changed slightly after radiolabelling with ^89^Zr‐oxine for 48 hours. In addition, the radiolabelling yield was promoted from 10% to 40% due to the increase in the amount of CAR T cells. Radiochemical purity was >95%.

### 3.3 Pharmacodynamic study

**FIGURE 2:**
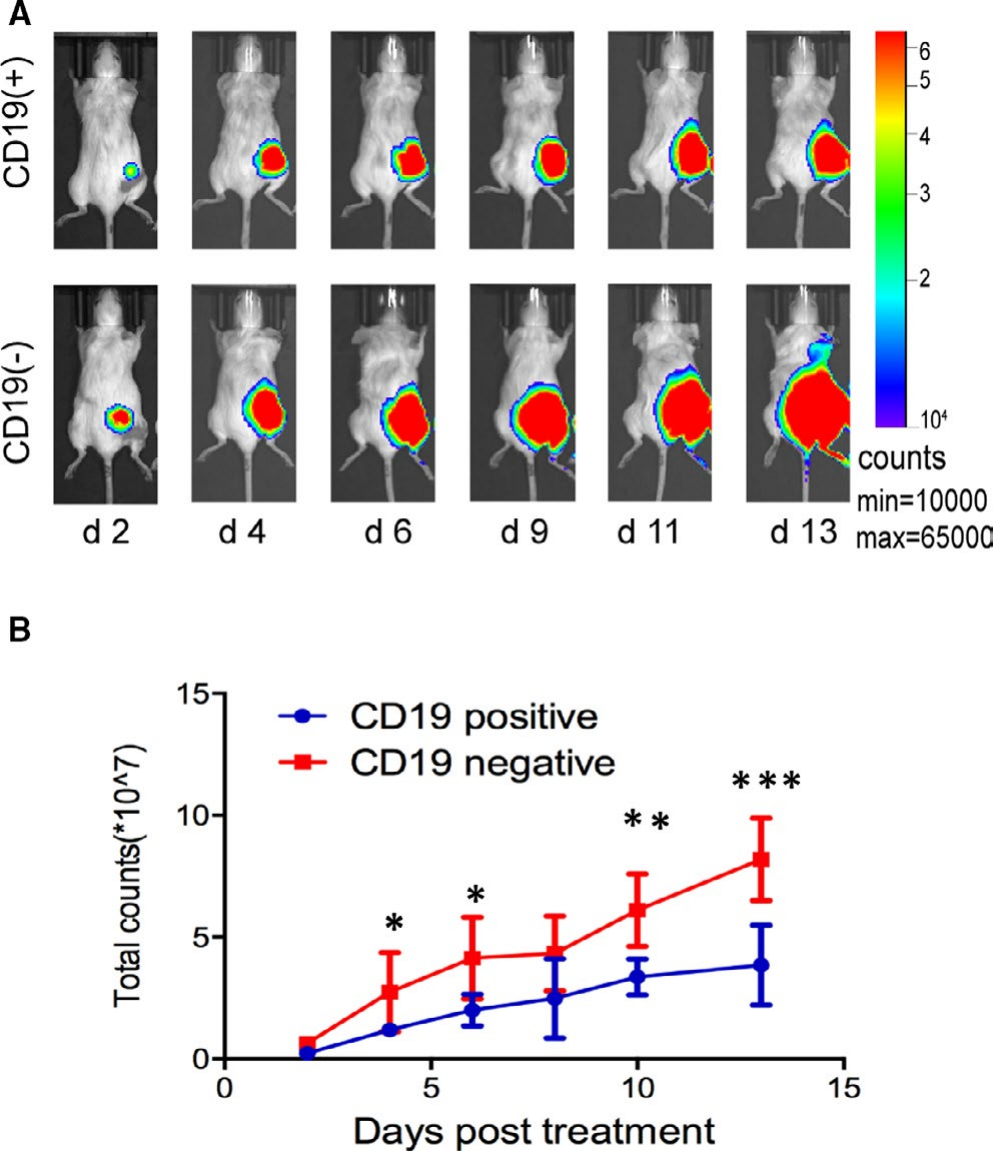
Pharmacodynamic study. A, Representative fluorescent imaging of CD19‐positive/negative tumours on days 2, 4, 6, 9, 11 and 13 after treatment with CD19 CAR T cells. B, Quantified fluorescent intensity in CD19‐positive/negative tumours was shown

The results of tumour cell activity evaluated with bioluminescence imaging are shown in Figure 2A,B. Obviously, the CD19‐positive group had a lower tumour cell fluorescent intensity than the CD19‐negative group during the entire treatment. At the early stage (day 4), the tumour fluorescent intensity of the CD19‐positive group (1.19 ± 0.20) was significantly lower than that of the CD19‐negative group (3.28 ± 1.03) with *P* = .011. In addition, the tumour suppressive effect was maintained until the monitoring end‐point, with the tumour fluorescent intensity values of 3.85 ± 1.64 for the CD19‐positive and 8.18 ± 1.68 for the CD19‐negative group on day 13 (*P* = .0005).

**FIGURE 3:**
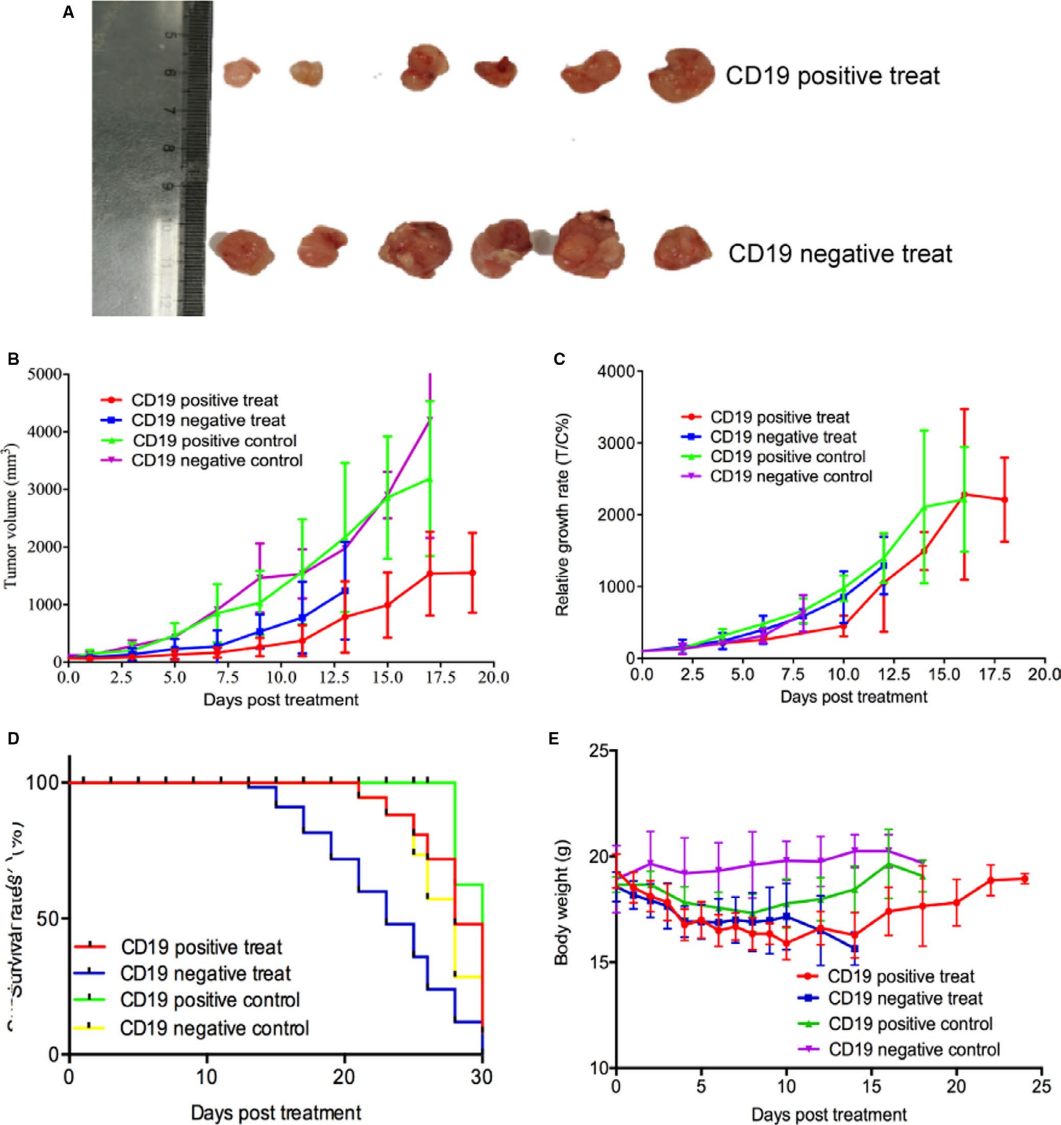
Observed profiles for in vivo CAR T cells induced tumour growth inhibition. A, Representative ex vivo images of tumours at 168 h after administration of CAR T cells. Treated and control tumour volumes are measured, and tumour growth curves (B) are plotted. Treated and control relative tumour growth rates (C) are also represented. (D) Survival rates of CD19‐positive/negative groups are shown. E, Bodyweights of the CD19‐positive/negative groups are shown

Also, from the photographs of tumours at 168 hours shown in Figure 3A, we can obviously find the difference in tumour size between the two groups. Tumour volume and tumour relative growth rate were analysed in Figure 3B, C. The results showed that the CD19‐positive group achieved much tumour inhibition by CAR T therapy, which is consistent with the results of bioluminescence imaging. To avoid the influence of initial tumour volume on the evaluation of efficacy, we calculated the relative growth rates of tumours in each group. On day 10, the relative growth rate of the CD19‐positive group was 450 ± 142 T/C%, while that of the CD19‐negative group (853 ± 360 T/C%) and CD19‐positive control group (978 ± 177 T/C%) was significantly high (*P* < .05). A survival study was performed as well (Figure 3D), and the results indicated that the CD19‐positive group died from day 17 until day 30 due to individual differences in therapy efficacy. In contrast, the CD19‐negative model mice died around day 15 due to ineffective treatment and adverse reactions. In addition, the two treated groups achieved bodyweight loss during 0‐15 days, and then, they gradually restored, while the bodyweight of the two control groups did not decrease during the whole experimental period (Figure 3E).

### 3.4 In Vivo PET Tracking of ^89^Zr‐Radiolabelled CAR T cells

**FIGURE 4:**
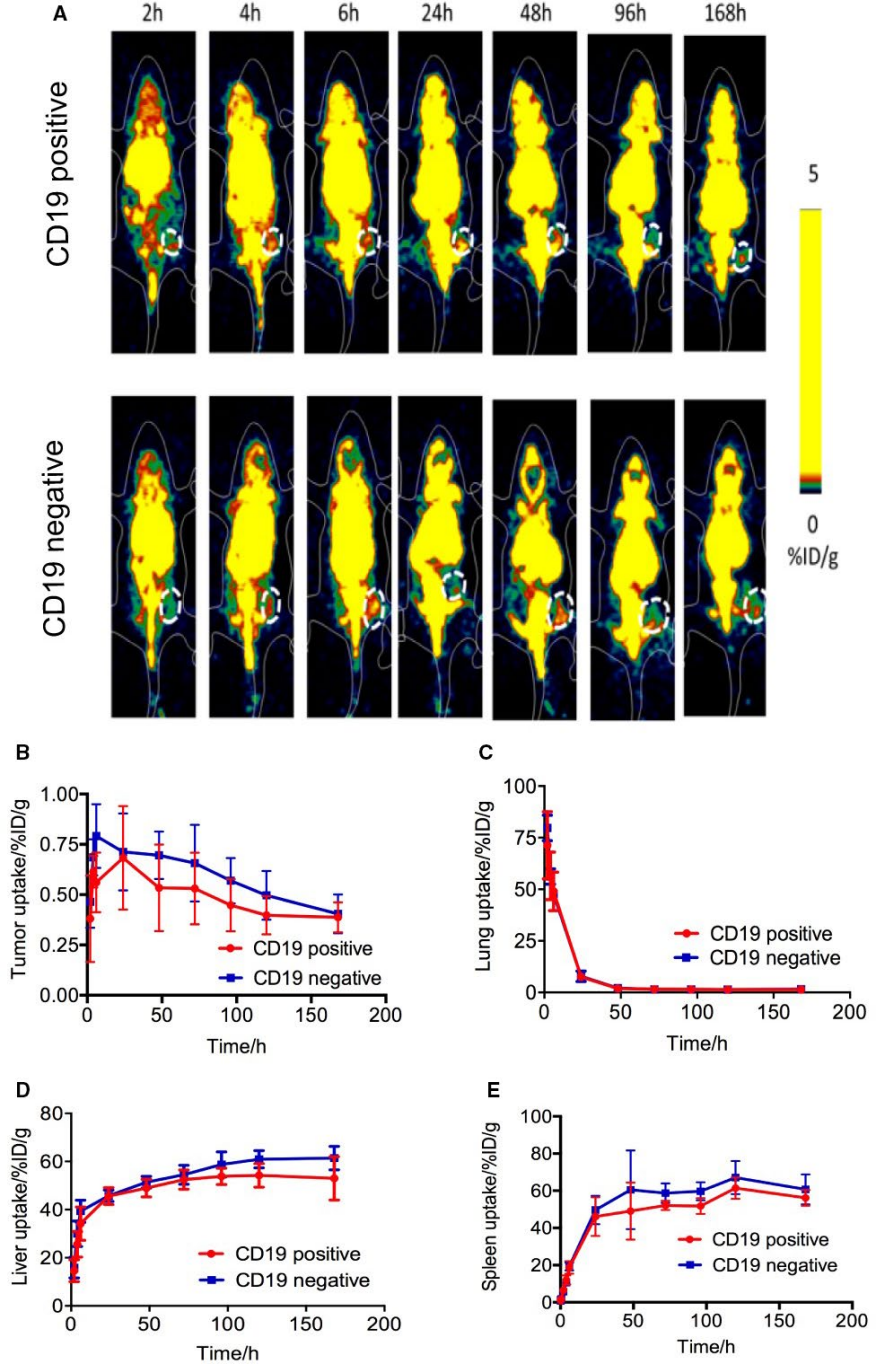
In vivo tracking of ^89^Zr‐CAR T cells in CD19‐positive/negative mice (n = 6) by static PET scan. A, Representative non‐invasive micro‐PET imaging of mice bearing tumours after administration of ^89^Zr‐labelled CAR T cells within 168 h. Time‐dependent ^89^Zr‐labelled CAR T‐cell uptake percentages are represented in tumour (B), lung (C), liver(D) and spleen(E) within 168 h

To further validate the tumour‐targeting performance of ^89^Zr‐labelled CD19 CAR T on CD19‐positive tumour in vivo, micro‐PET imaging was performed in the CD19‐positive group and CD19‐negative group. Tomographic images of multiple time‐points after intravenous injection are shown in Figure 4A. The uptake (%ID/g) of tumour, lung, liver and spleen is shown in Figure 4B. Consistent with the results of our previous study,^27^ in mice of two groups, ^89^Zr‐labelled CAR T cells were distributed in the lungs at 2 hours (71.36 ± 16.27%ID/g for the CD19‐positive and 79.74 ± 6.19%ID/g for the CD19‐negative group) and then rapidly decreased to 1.37 ± 0.29%ID/g (CD19‐positive group) and 1.60 ± 0.20%ID/g (CD19‐negative group) for the next 7 days. The ^89^Zr‐CAR T cells were distributed in the liver (45.63 ± 3.54%ID/g for the CD19‐positive and 45.75 ± 2.35%ID/g for the CD19‐negative group) and spleen (46.07 ± 10.43%ID/g for the CD19‐positive and 49.65 ± 7.67%ID/g for the CD10‐negative group) at 24h and maintaining a plateau (Figure 4C, D, E). No significant changes appeared in tumour uptake during the long‐term monitoring, and on day 7, the uptake of tumour in CD19‐positive tumour and CD19‐negative tumour was 0.39 ± 0.07%ID/g and 0.40 ± 0.10%ID/g, respectively (*P* > .05).

### 3.5 Ex Vivo Biodistribution of ^89^Zr‐Radiolabelled CAR T cells

**FIGURE 5:**
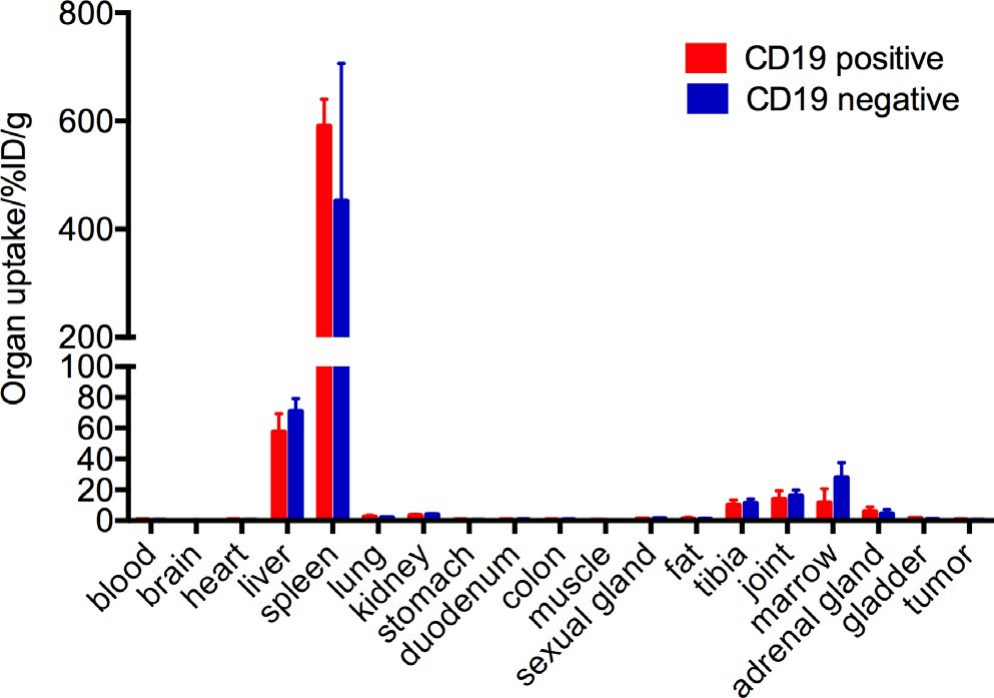
Biodistribution of ^89^Zr‐labelled CAR T cells in major organs at 96 h after administration of ^89^Zr‐labelled CAR T cells

Ex vivo biodistribution of ^89^Zr‐labelled CAR T cells at 168 hours post‐injection of radiolabelled CAR T cells is shown in Figure 5, which revealed a high concentration of radiolabelled CAR T cells in spleen (590.58 ± 49.36%ID/g, n = 6) and liver ( 57.43 ± 11.86%ID/g, n = 6), followed by joint (13.84 ± 5.44%ID/g, n = 6) and marrow (11.70 ± 8.93%ID/g, n = 6) in the CD19‐positive group, and the same is observed in the CD19‐negative group, with spleen (452.48 ± 253.79%ID/g, n = 6), liver (70.94 ± 8.36%ID/g, n = 6), joint (16.08 ± 3.88%ID/g, n = 6) and marrow (27.79 ± 9.88%ID/g, n = 6). The tumour uptake (%ID/g) in the CD19‐positive group was 0.45 ± 0.28%ID/g and 0.32 ± 0.16%ID/g in the CD19‐negative group. Uptake of ^89^Zr‐CAR T cells in tumours showed no major differences between the two groups. Compared with uptake calculated by PET image, there is no significant difference between all the organs except spleen. We speculate that this is because the spleen is so small that the uptake values of PET imaging were susceptible to boundary volume effects.

### 3.6 Pharmacokinetics

**FIGURE 6:**
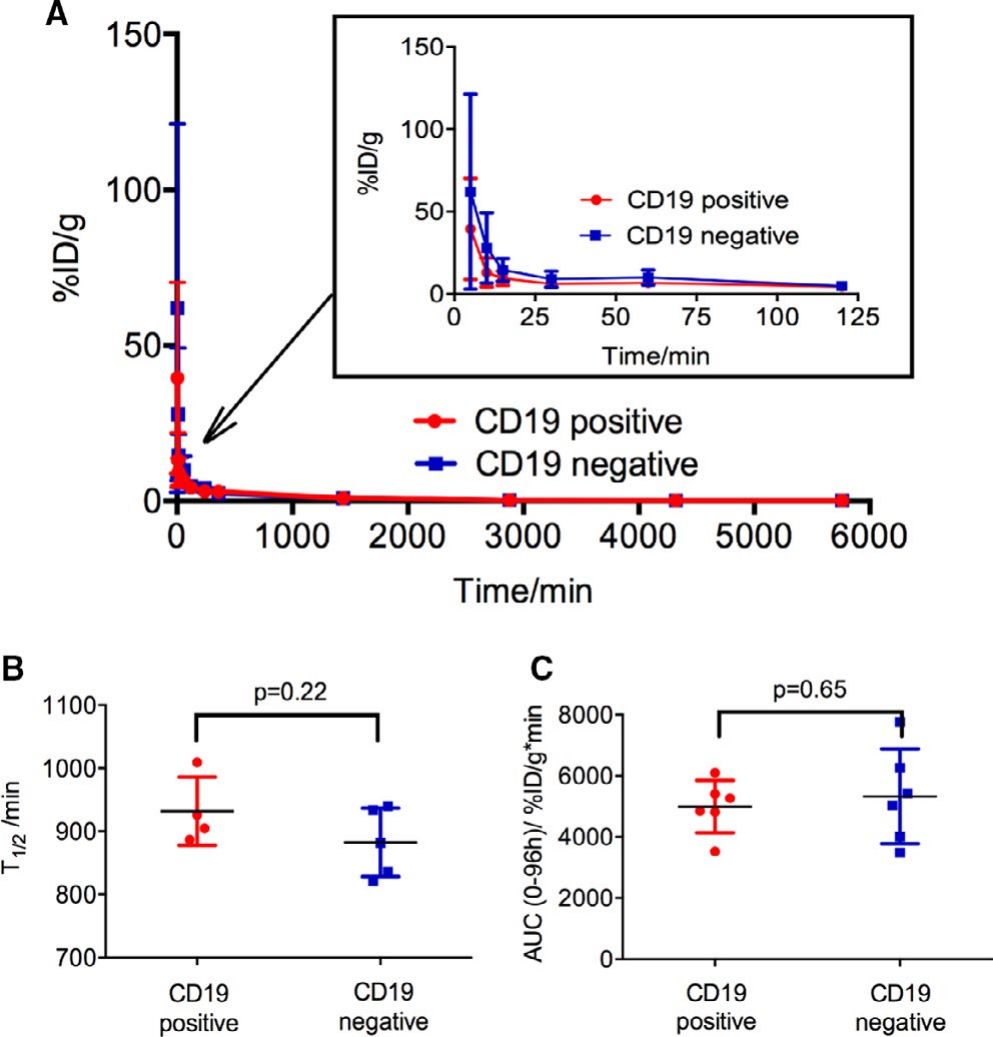
Pharmacokinetics of ^89^Zr‐labelled CAR T cells over time. A, The curve of blood uptake of ^89^Zr‐labelled CD19 CAR T cells in two groups within 96 h. B, The AUC (0‐96 h) of the blood pharmacokinetics of ^89^Zr‐CAR T cells calculated by DAS. C, The mean half‐life (*t*_1/2_) of ^89^Zr‐CD19 CAR T cells in two groups

As shown in Figure 6A, the curve of blood uptake of ^89^Zr‐labelled CD19 CAR T cells in two groups was fitted with DAS. At 5 minutes after injection, the blood uptake was 16.59 ± 6.46%ID/g in the CD19‐positive group and 20.62 ± 16.54%ID/g in the CD19‐negative group and then rapidly decreased below 1%ID/g within one day. The AUC(0‐96 hours) of the blood pharmacokinetics of ^89^Zr‐CAR T cells was 4991.82 ± 857.76 in the CD19‐positive mice and 5328.47 ± 1550.54 in the CD19‐negative mice with no differences (*P* > .05) (Figure 6B). The mean half‐life(*t*_1/2_) of ^89^Zr‐labelled CD19 CAR T cells in the CD19‐positive mice and CD19‐negative mice was 931.77 ± 54.11 minutes and 882.51 ± 54.25 minutes, respectively (*P* > .05) (Figure 6C).

### 3.7 Specific tumour uptake

**FIGURE 7:**
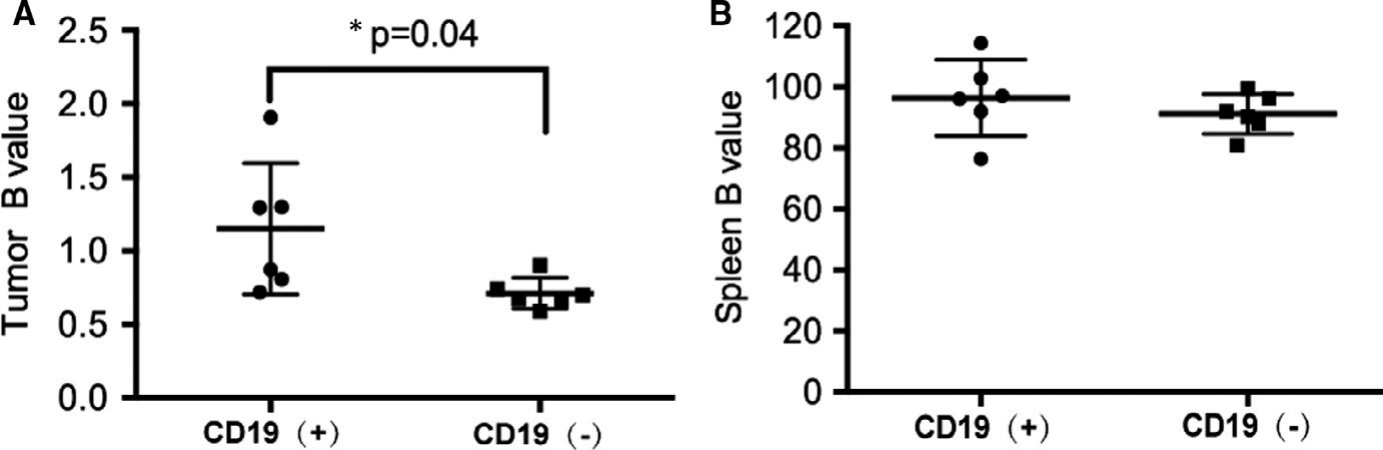
Specific uptake at 96 h after administration of ^89^Zr‐labelled CAR T cells in tumour (A) and spleen (B)

The B‐value of tumour‐specific uptake between CD19‐positive and CD19‐negative groups is shown in Figure 7, which suggested a higher degree of specific binding to ^89^Zr‐labelled CAR T cells in CD19‐positive tumour tissues. The B‐value was 1.15 ± 0.45 in CD19‐positive mice and 0.71 ± 0.11 in CD19‐negative mice with *P* = .04.

## 4 DISCUSSION

In this study, CD19 CAR T cells targeting validation in vitro and killing function, cell proliferation, cytokine release after antigen stimulation and so on were investigated. It demonstrates that CD19 CAR T cells specifically target CD19‐K562 cells. Therefore, in order to verify the in vivo CD19 targeting of CAR T cells, CD19‐positive and CD19‐negative solid tumour models were established using CD19‐K562‐Luc and K562‐Luc cell lines, respectively.

Consistent with previous reports,^27^ CD19 CAR T cells can be successfully labelled with ^89^Zr without affecting their viability and proliferation, and the specific activity can meet the requirements for long‐term in vivo monitoring. We used PET and optical imaging to investigate the PK and PD performance of CD19 CAR T cells in CD19 high expression solid tumours, respectively. PET imaging showed the distribution and migration of CAR T cells in tumour‐bearing mice. Although no visual radioactivity difference appeared between CD19‐positive and negative tumours, Patlak modelling analysis revealed the specific uptake of CAR T cells targeting CD19 in CD19‐positive solid tumours compared with CD19‐negative tumours. As far as we know, this is the first time using a mathematical model to verify the CD19 targeting with ^89^Zr‐labelled CAR T cells using PET imaging in solid tumour models. Due to the abundant blood vessels in tumours and the retention characteristic of ^89^Zr nuclide, ^89^Zr‐labelled CAR T‐cell exposure in blood can elevate tumour tissue uptake, so PK behaviour in vivo should be considered for tumour specificity analysis. This result was consistent with the PD results by the fluorescent imaging and tumour volume.

The signal of PET imaging of CAR T cells directly labelled with ^89^Zr‐oxine only reflects the distribution and migration of injected cells and does not account for cell proliferation, which might initiate the majority of the antitumour response. Therefore, although the increase in CAR T‐cell uptake in CD19‐positive tumours is very limited compared with CD19‐negative tumours, CAR T cells can survive longer in CD19‐positive tumours due to their ‘live’ nature, resulting in a dramatic difference in efficacy between the two groups, which demonstrates the magic of CAR T‐cell immunotherapy.

Currently, we can monitor the clinical efficacy of CAR T therapies by performing direct peripheral blood tumour‐specific T‐cell counts, serum analysis of cytokines associated with T‐cell activation and (repeat) tumour biopsies. In this experiment, we also tried to use the qPCR method to measure DNA copy number in blood samples. This method has two drawbacks: first, invasive collection of at least 100 µL of blood for qPCR analysis is difficult to consecutively obtain in small animal models, while PET is a non‐invasive and real‐time dynamic monitoring; second, the distribution of CAR T cells in all normal tissues throughout the body cannot be examined to assess their safety; and furthermore, in this study, the test results of qPCR were below the detection limit regardless of the efficacy, indicating that the sensitivity of this method might be inferior to PET.

In addition to the difficulty of CAR T cells to migrate inside solid tumours, another major challenge to the efficacy of solid tumours is the selection of targets.^30^ The effectiveness of CAR T therapy is closely related to targeting. Our preliminary PK and PD results suggest that PET imaging using ^89^Zr‐labelled CAR T cells can be used for the targeting validation of CAR T cells, and is expected to be used for subsequent CAR T‐cell target screening and efficacy prediction.

Nevertheless, the cell tracking method in this study has certain limitations: on the one hand, direct labelling CAR T cells can only reveal the distribution, migration and homing of parental cells in vivo, and cannot show the cell proliferation, activation or death; on the other hand, we did not further validate this finding in a Raji xenograft model. The selection of animal models is particularly important when conducting CAR T therapy PK/PD studies as a suitable animal model can mimic the human in vivo tumour microenvironment and reflect drug targeting on CAR T‐cell mechanism of action. Subsequently, we plan to further validate this PK/PD result in situ haematoma animal models.

In summary, we used PET and optical imaging to investigate the PK and PD of CD19 CAR T‐cell target therapy for leukaemic solid tumour. CD19‐positive solid tumours had specific targeting uptake and better tumour suppression efficiency after CD19 CAR T treatment. This approach is promising to provide a basis for PK‐PD investigation and deserves further evaluation of CAR T behaviour on various cell types and animal models.

## CONFLICT OF INTEREST

The authors declared no potential conflicts of interest.

## AUTHOR CONTRIBUTIONS

**Qiong Wu:** Data curation (equal); Visualization (lead); Writing‐original draft (equal). **Yan Wang:** Data curation (equal); Writing‐original draft (equal); Writing‐review & editing (supporting). **Xinyu Wang:** Conceptualization (supporting); Supervision (equal); Writing‐review & editing (supporting). **Ningxia Liang:** Data curation (equal); Supervision (equal). **Jingjing Liu:** Data curation (equal); Formal analysis (equal). **Donghui Pan:** Data curation (equal); Supervision (equal). **Yuping Xu:** Data curation (equal); Supervision (equal). **Lizhen Wang:** Supervision (supporting). **Junjie Yan:** Supervision (supporting). **Guangji Wang:** Funding acquisition (equal). **Liyan Miao:** Funding acquisition (supporting). **Min Yang:** Conceptualization (lead); Funding acquisition (equal); Project administration (lead); Resources (lead); Supervision (lead); Writing‐review & editing (lead).

## Data Availability

The data that support the findings of this study are available from the corresponding author upon reasonable request.
